# Comparative Effects of Dexmedetomidine and Propofol on US-Guided Radiofrequency Ablation of Hepatic Neoplasm Under Monitored Anesthesia Care: A Randomized Controlled Study

**DOI:** 10.1097/MD.0000000000001349

**Published:** 2015-08-14

**Authors:** Kyoung-Woon Joung, Seong-Soo Choi, Dong-Min Jang, Yu-Gyeong Kong, Hwa-Mi Lee, Ji-Hoon Shim, Hyung-Jin Won, Yong-Moon Shin, Pyo-Nyun Kim, Myung-Hee Song

**Affiliations:** From the Department of Anesthesiology and Pain Medicine (K-WJ, S-SC, D-MJ, Y-GK, J-HS, M-HS), Asan Medical Center, University of Ulsan College of Medicine, Seoul; Department of Anesthesiology and Pain Medicine (H-ML), Gangneung Asan Hospital, University of Ulsan College of Medicine, Gangneung; and Department of Radiology (H-JW, Y-MS, P-NK), Asan Medical Center, University of Ulsan College of Medicine, Seoul, Korea.

## Abstract

Percutaneous radiofrequency ablation (RFA) is a useful and safe procedure for treating hepatic neoplasm. However, liver RFA causes severe pain, which thereby increases the demand for monitored anesthesia care (MAC). Here, we compared the efficacy and safety of propofol and dexmedetomidine, which are commonly administered during MAC when performing RFA to assess hepatic neoplasm.

In this randomized controlled trial, 40 patients were randomly allocated to 2 groups for elective RFA. Patients received either dexmedetomidine (group D) or propofol (group P). Both groups received the continuous infusion of remifentanil for pain control. The primary outcomes were opioid consumption and differences in partial pressure of arterial carbon dioxide (PaCO_2_) between pre- and postprocedure RFA. In addition, hemodynamic parameters, patient satisfaction, and interventional radiologist satisfaction were determined.

There were significant differences in opioid consumption (50.1 ± 16.8 ng/kg/min [group D] vs 71.2 ± 18.7 ng/kg/min [group P]; *P* = 0.001) and delta PaCO_2_ (10.4 ± 6.4 mm Hg vs 17.2 ± 9.2 mm Hg, respectively; *P* = 0.016). Moreover, respiratory rates were significantly different between groups during RFA (*P* < 0.001). However, blood pressure and heart rate did not significantly change during RFA. Neither patient nor interventional radiologist satisfaction was significantly different between groups.

Dexmedetomidine provides better respiratory stability and reduces opioid consumption in comparison with propofol when administered under MAC when performing RFA for hepatic neoplasm.

## INTRODUCTION

Percutaneous radiofrequency ablation (RFA) is an accepted, useful, and safe procedure for treating benign and malignant neoplasms in various organs such as the lung, kidney, bone, and liver.^1–5^ Most importantly, the usefulness of RFA has emerged as a treatment modality for treating liver neoplasm because RFA is less invasive and reduces the length of hospital stay more than surgical resection. Moreover, RFA has been performed as an alternative treatment for unresectable primary hepatocellular carcinoma, which often occurs in hepatic reserve-impaired cirrhotic liver.^6^

Patients who receive RFA usually complain of severe pain during the procedure, but the interventional radiologist typically requires the patients to cooperate in order to determine the tumor location and precise RFA performance. Hence, the demand of monitored anesthesia care (MAC) during RFA has increased. Propofol is commonly used sedative drug for various procedures under sedation^7,8^ because it shows fast onset, short half-life, and rapid recovery. However, propofol has a serious problem as severe respiratory depression and even apnea. On the contrary, dexmedetomidine—the highly selective alpha 2 agonist—demonstrates both analgesic and hypnotic properties without respiratory depression and also reduces stress responses to surgery by reducing sympathetic activity.^9^ For these reasons, dexmedetomidine is commonly used in intensive care units as a sedative. After considering the pharmacologic profiles of these drugs, we hypothesized that, in comparison with propofol, dexmedetomidine will provide safer and better sedation under MAC when performing RFA.

## METHODS

### Study Population

We obtained approval from the Institutional Review Board of our institution (2013-0797) and written informed consent from all patients before starting this prospective, randomized, controlled study. Between September 1, 2013 and January 3, 2014, we enrolled physical status I to II patients (according to American Society of Anesthesiologists criteria), who were >20 years of age and scheduled to receive elective percutaneous RFA under MAC to treat single hepatic tumors. Patients with chronic obstructive pulmonary disease, renal failure, cardiac disease, cerebrovascular disease, negative modified Allen test, contraindications for dexmedetomidine or propofol, or history of emergency RFA were excluded. This study was also registered at http://cris.nih.go.kr (KCT0000838).

Patients were randomly allocated to 2 groups using computer-generated codes that were maintained in sequentially numbered opaque envelopes. On the morning of intervention before inducing anesthesia, allocation envelopes were opened by a nurse or anesthesiologist in a blind manner who then prepared either propofol or dexmedetomidine for continuous infusion. None of the other anesthesiologists involved in patient management or data collection were aware of the group assignment.

### Anesthetic Management

All patients fasted for ≥8 hours and were not premedicated. Anesthetic monitoring includes noninvasive blood pressure, pulse oximetry, electrocardiography, bispectral index (BIS), and capnography. All patients received 6 L oxygen using a simple facemask. In the dexmedetomidine group (group D), MAC was induced with the loading dose of dexmedetomidine (1 μg/kg) over 15 minutes. The maintenance dexmedetomidine dose was adjusted to the appropriate sedation criteria for RFA (0.1–0.2 μg/kg/h). In the propofol group (group P), MAC was induced and maintained via the continuous infusion of propofol using a target-controlled infusion (TCI) pump. To determine the proper depth of sedation, the effect-site propofol concentration was adjusted using steps of 0.1 to 0.2 μg/mL. In both groups, the continuous infusion of remifentanil using a TCI pump was simultaneously performed during infusion with dexmedetomidine or propofol. The remifentanil dose was adjusted to maintain the mean blood pressure to within 20% of the baseline (using 0.5 ng/mL steps). The appropriate level of sedation for liver RFA was 3 points on the Modified Observer's Assessment of Alertness/Sedation scale and 65 to 80 points on BIS. At this level of sedation, patients seemed comfortable, lost consciousness, and maintained spontaneous breathing. However, when the interventionist or anesthesiologist requested patient cooperation, the patient immediately became alert and followed the request. We defined hypoxia as peripheral oxygen saturation <90%, and applied the triple airway maneuver to maintain the airway at any time during the procedure. All anesthetic drugs were discontinued immediately after RFA.

### RFA for Hepatic Neoplasm

Our ultrasound-guided percutaneous RFA technique has been previously described in detail.^10^ In brief, tumor ablation was performed by 1 of 3 interventional radiologists with >5 years of experience in a blind manner. We used single electrodes with an internally cooled tip (Cool-tip™; Covidien, Burlington, MA), cooled wet tip (Jet-tip^®^, RF Medical Co., Ltd., Seoul, Korea), and multitined expandable tip (Proteus^®^, STARmed Co., Ltd., Goyang, Korea), as appropriate. The RFA current was elevated 20 W/min starting from 60 W with internally cooled tip and multitined expandable tip, or 30 W/min starting from 50 W with wet tip using the automatic impedance control method and 200-W generator (Mygen M-2004 Radiofrequency System; RF Medical Co.) for 8 to 18 minutes.

### Data Collection and Outcome Evaluation

Preprocedural clinical data were collected for all patients using our computerized patient record system (Asan Medical Center Information System Electronic Medical Record). Collected data included demographics, comorbidities, preoperative Model for End-Stage Liver Disease score, reason for RFA, tumor location, tumor size, and preoperative arterial blood gas analysis (ABGA) data. Intraprocedural data were also collected, including noninvasive blood pressure, heart rate (HR), respiration rate, BIS value, procedure time, anesthetic time, use of artificial ascites technique, type of ablation tip, maximal energy, total remifentanil dose, and postprocedural ABGA. Intraprocedural vital signs were measured and recorded at 3-minute intervals. ABGA was performed at the end of the procedure. Iowa Satisfaction with Anesthesia Scale (ISAS) was assessed the day following the procedure. ISAS is known as a reliable, valid, and useful questionnaire for measuring patient satisfaction with MAC.^11^ Interventionist satisfaction during the procedure was assessed according to the 7-point Likert scale (7 = best; 6 = better; 5 = good; 4 = not good, but not bad; 3 = bad; 2 = worse; 1 = worst).

The primary outcomes of this study were opioid consumption and pre- and postprocedural differences in partial pressure of arterial carbon dioxide (PaCO_2_). The secondary outcomes included changes in vital signs and BIS values during the procedure, interventionist satisfaction, and ISAS score after RFA.

### Statistical Analysis

Based on 20 preliminary patients (10 patients in each group) who demonstrated PaCO_2_ differences (21.67 mm Hg in group P vs 11.24 mm Hg in group D), a sample size of 17 patients per group was calculated in order to obtain 80% statistical power at a significance level of 0.05 (2-tailed). To allow for 15% loss during the study period, we intended to recruit a total of 40 patients.

Continuous variables are presented as the mean ± SD or median with the interquartile range, and the categorical variables are presented as number of patients and percentages. Continuous data were analyzed using the *t* test or Mann–Whitney rank-sum *U* test, and categorical data were tested using the Pearson χ^2^ or Fisher exact test, as appropriate. Repeated measures data were analyzed using repeated measures analysis of variance (ANOVA) with post hoc analysis. Statistical analyses were performed using SPSS (version 21.0; SPSS Inc, Chicago, IL). For all comparisons, *P* < 0.05 is considered statistically significant.

## RESULTS

**FIGURE 1:**
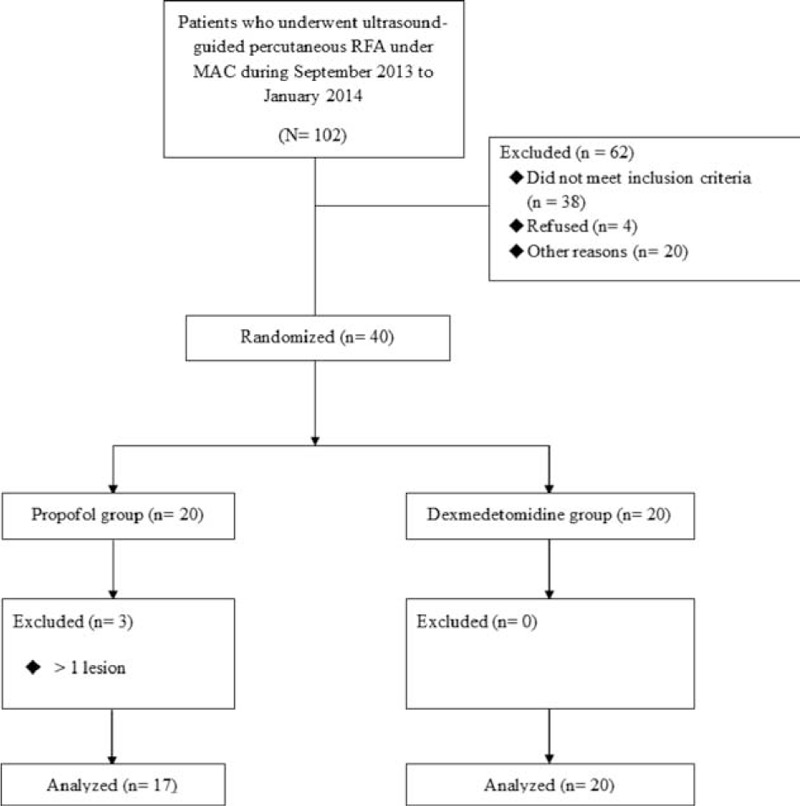
Study design according to the CONSORT statement. MAC = monitored anesthesia care, RFA = radiofrequency ablation.

Between September 2013 and January 2014, 40 patients were enrolled in this prospective, randomized, controlled study. All patients were randomly allocated to groups P or D. Among the initially enrolled patients, 3 patients were excluded because of newly identified multiple tumors and ablated >1 lesion (all these patients were enrolled in group P). Therefore, 37 patients were included in the final analysis (Figure 1). In the final analysis, groups P and D consisted of 17 and 20 patients, respectively.

**FIGURE 2:**
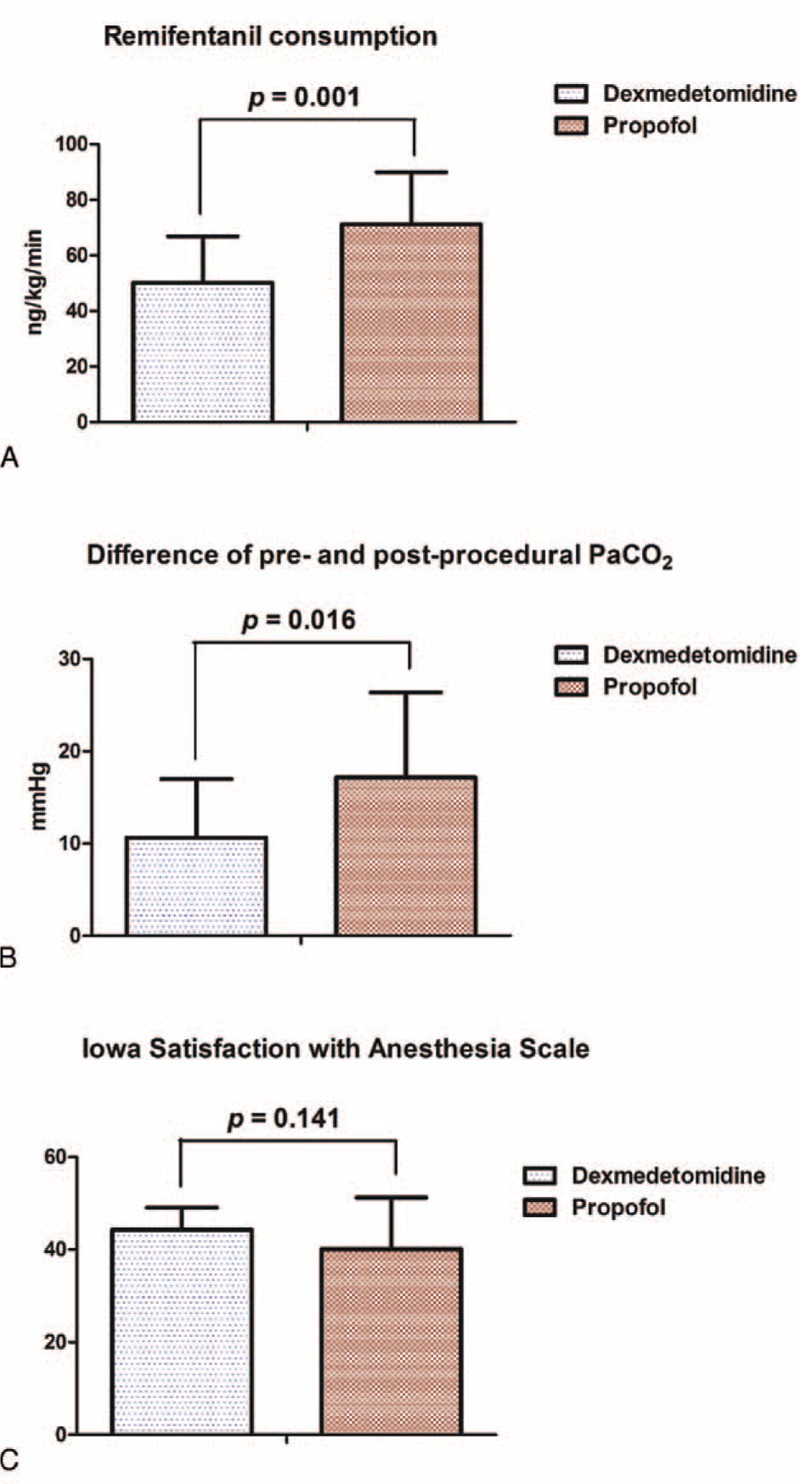
Differences in primary outcomes and Iowa Satisfaction with Anesthesia Scale. Data are expressed as the mean ± SD. PaCO_2_ = partial pressure of arterial carbon dioxide.

There were no significant differences in terms of demographic data or technical RFA details between groups (Tables 1 and 2). There were significant differences between groups in terms of opioid consumption and pre- and postprocedural PaCO_2_. Remifentanil consumptions in groups D and P during RFA were 50.1 ± 16.8 and 71.2 ± 18.7 ng/kg/min, respectively (*P* = 0.001). Differences in pre- and postprocedural PaCO_2_ were 10.6 ± 6.3 and 17.2 ± 9.2 mm Hg, respectively (*P* = 0.016). However, ISAS scores were not significantly different between groups: 44.3 ± 4.8 in group D versus 40.1 ± 11.1 in group P (*P* = 0.141) (Figure 2).

**TABLE 1:**
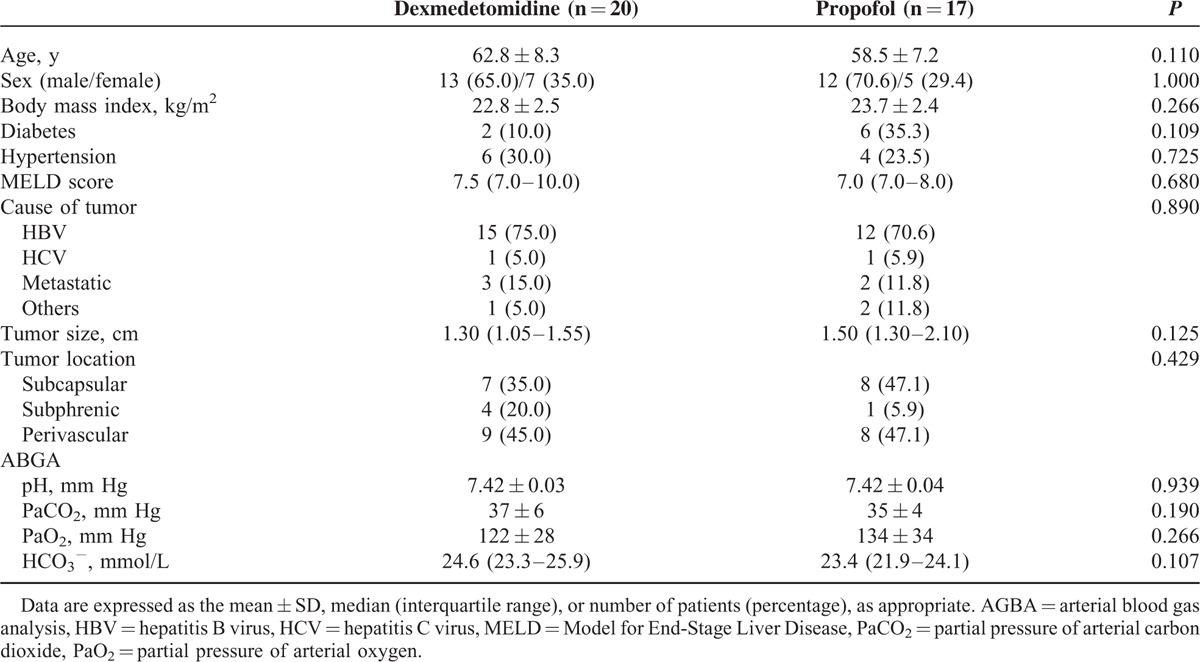
Baseline Characteristics and Intraprocedural Variables

**TABLE 2:**
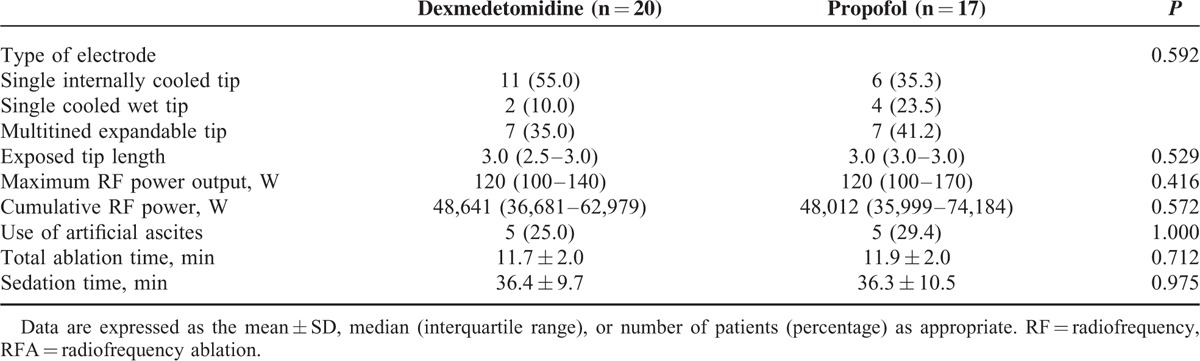
Technical Details of the RFA Procedure

**FIGURE 3:**
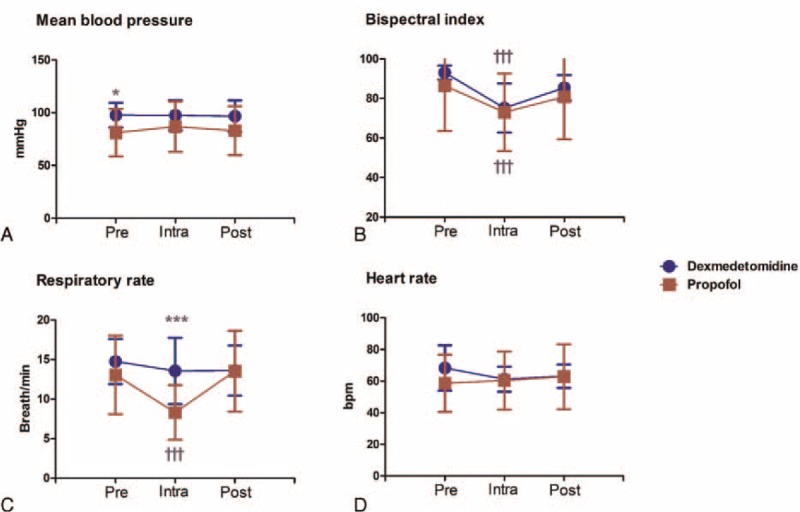
Changes in vital signs and BIS values during radiofrequency ablation. Data are expressed as the mean ± SD during radiofrequency ablation of hepatic neoplasm. ∗*P* < 0.05 and ∗∗∗*P* < 0.001 when compared between groups. †††*P* < 0.001 compared with preprocedure. BIS = bispectral index, Intra = intraprocedure, Post = postprocedure, Pre = preprocedure.

As shown in Figure 3A, repeated measures ANOVA indicated a significant difference between groups in terms of mean blood pressure (MBP) (F = 10.753; *P* = 0.001). However, post hoc MBP analysis showed a significant difference only in terms of preprocedure (*P* = 0.015), but not intra- (*P* = 0.163) or postprocedure MBP (*P* = 0.076). Repeated measures ANOVA indicated no significant differences between groups in terms of BIS (F = 0.422; *P* = 0.517; Figure 3B). Post hoc analysis of BIS showed significant differences between both groups in terms of pre- and intraprocedural BIS (*P* < 0.001 for groups D and P) and intra- and postprocedure BIS (*P* < 0.001 for group D; *P* = 0.01 for group P).

Repeated measures ANOVA indicated significant differences between groups in terms of the respiratory rate (RR) (F = 5.936; *P* = 0.017; Figure 3C). Post hoc analysis showed a significant reduction of RR in group P in comparison with group D during the procedure (*P* < 0.001), but not pre- or postprocedure (*P* = 0.509 and 0.541, respectively). Furthermore, in group P, RR was lower during the procedure in comparison with preprocedure. As shown in Figure 3D, no significant differences were noted in terms of HR in both groups. In addition, all interventional radiologists were satisfied with MAC during RFA (satisfaction scale = 7), and all patients were awake and fully recovered by the end of procedure. No postprocedural complications were observed.

## DISCUSSION

MAC is an alternative anesthetic technique for anesthesiologist monitoring during interventional procedures (with or without sedation). Generally, the ideal patient is spontaneously breathing and feeling comfortable and cooperative. Thus, the agent used in MAC must demonstrate minimal side effects on cardiovascular, respiratory, and neurological systems.^12^ In this context, the results of this study suggest that dexmedetomidine may be more suitable than propofol for use in MAC during RFA because fewer opioids are consumed and less carbon dioxide (CO_2_) is retained without significantly changing MBP or HR during the procedure, although sedation was adequately maintained in both groups and ISAS did not differ between groups.

Remifentanil consumption was lower in group D than group P during RFA. These findings are important for several reasons. It is well known that opioid-induced respiratory depression is the most serious adverse effect of these drugs. Opioids induce dose-dependent respiratory depression by directly acting on the respiratory centers in the brainstem.^13–15^ The RR is usually drastically slower following opioid overdose.^15^ In addition, Sarton et al^16^ reported that CO_2_-induced ventilatory stimulation is significantly reduced by opioids. High-dose opioids can also induce chest wall rigidity and ineffective ventilation.^17–19^ Although chest wall rigidity demonstrates a very low incidence, it causes unnecessary intubation and might be associated with poor outcomes. Moreover, high-dose remifentanil increases the risk of acute opioid tolerance and hyperalgesia. For example, Guignard et al^20^ have reported that relatively large-dose intraoperative remifentanil increases postoperative pain and the consumption of rescue drugs. In group D, which consumed fewer opioids in the present study, RR was maintained and the differences in pre- and postprocedural PaCO_2_ increased to a lesser extent than group P.

Clinically, hypoxia is a more significant problem than hypercapnia.^21^ We supplied oxygen to all our patients to prevent hypoxia. In this condition, hypoventilation and CO_2_ retention are more important because patients can develop respiratory depression despite normal oximetry readings.^22^ As described earlier, the best benefit of using dexmedetomidine for sedation is its small effect on ventilation.^23^ Here, group D demonstrated lower CO_2_ retention than group P without significantly changing RR.

There are some limitations to this study. First, our observations were performed in a double-blind manner because of the differences in the nature of each drug, including delivery system and pain on intravenous injection. The TCI pump is a computer-assisted drug delivery system that predicts the plasma and effect-site concentration without complex calculations.^24^ Propofol is widely used with TCI modeling because of its well-known plasma-effect-site equilibration constant (Ke_0_). However, dexmedetomidine is not currently used with TCI because the Ke_0_ value of dexmedetomidine has not been widely studied. Second, this study had a relatively small sample size, although we calculated the sample size according to the results of a pilot study. Here, MBP was significantly different between 2 groups. However, our post hoc analysis only showed preprocedural differences in MBP. This baseline difference in MBP was, in part, caused by the enrollment of more severe hypertensive patients in group D than in group P, although no statistical significance was observed. If we used a larger sample size, the baseline differences in the MBP might decrease.

In conclusion, dexmedetomidine provides better respiratory stability and reduces opioid consumption in comparison with propofol when administered under MAC when performing RFA for hepatic neoplasm. For safer sadation of patients, we recommend dexmedetomidine when used in MAC during RFA for hepatic neoplasm than propofol.
